# Genome Sequence of Influenza B Epidemic Strain B/Almaty/8/2018

**DOI:** 10.1128/mra.00544-22

**Published:** 2022-09-22

**Authors:** Aidyn Kydyrmanov, Kobey Karamendin, Nailya Klivleyeva, Temirlan Sabyrzhan, Bibiazhar Suleimen, Eldar Ismailov, Tatyana Glebova

**Affiliations:** a Research and Production Center for Microbiology and Virology, Almaty, Kazakhstan; Queens College CUNY

## Abstract

An influenza virus strain, B/Almaty/8/2018, was isolated in Almaty (in southeastern Kazakhstan) during a human population surveillance study in 2018. Here, we present the nearly complete genome sequence of this epidemic strain, compared to the Yamagata-like and Victoria-like variants of the influenza B virus.

## ANNOUNCEMENT

Influenza B viruses are single-stranded segmented negative-sense RNA viruses belonging to the genus *Betainfluenzavirus* in the family *Orthomyxoviridae* (1). Contemporary epidemic influenza B viruses are phylogenetically and antigenically divided into Yamagata-like and Victoria-like evolutionary lines (2). The type B virus caused a substantial proportion of influenza infections globally in the 21st century, and its two lineages differed in terms of age and geographical distribution of patients. A lineage-level vaccine mismatch was observed in over 40% of seasons in temperate countries and 30% of seasons in the tropics (3).

During a seasonal influenza epidemic in Almaty, Kazakhstan, the strain B/Almaty/8/2018 was isolated from an oropharyngeal swab sample from an elderly patient with clinical symptoms of respiratory disease (4). This virus was isolated by inoculating samples into 10-day-old embryonated chicken eggs, with 48 h of incubation at 35°C (5). A hemagglutination test with chicken erythrocytes was performed to detect the virus. Viral RNA was extracted using the QIAamp viral RNA minikit (Qiagen). Next-generation sequencing determined its sequence using the NEBNext RNA sequencing kit and the rRNA depletion kit (New England Biolabs, USA). Paired-end sequencing of multiple pooled samples was performed on a MiSeq instrument, using the MiSeq reagent kit v2 (Illumina).

In total, 3,664,950 raw sequencing reads per sample were obtained, with a mean length of 250 nucleotides per read. The sequence data obtained were trimmed at the 3′ and 5′ ends with an error probability limit of 0.05, assembled, and mapped with the installed Geneious mapper (medium sensitivity, four iterations) against the eight segments of the reference strain В/Yamagata/16/1988 (GenBank accession numbers CY018765 to CY018772) using the Geneious v11.0 software (Biomatters) with default parameters. The final assembly of B/Almaty/8/2018 obtained with Geneious v11.0, which was 10,701 nucleotides in length, was aligned to the reference sequences of all eight gene segments of strain В/Yamagata/16/1988. BLASTn analyses of the obtained genome confirmed that it was closely related to influenza B viruses.

The gene coverage of the studied virus ranged from 73 to 2,760 nucleotides, while the average coverage was 1,300 nucleotides. At the nucleotide level, the hemagglutinin gene was 99% similar to that of viral strains isolated in 2017 in North America (Table 1). The data presented in Table 1 suggest the occurrence of reassortments of gene segments among influenza B strains.

**TABLE 1:** Comparison of the nucleotide sequences of all genes of the Kazakhstan influenza B strain with the genetically most closely related strains in GenBank

Gene or segment	Size (nucleotides)	GC content (%)	Most closely related strain	Identity with most closely related strain at nucleotide level (%)	GenBank accession no.
PB2	2,362	38.1	B/California/104/2015	99.79	KU592224.1
PB1	2,340	38.8	B/Wisconsin/18/2016	99.91	KX920685.1
PA	2,276	38.0	B/South Carolina/11/2016	99.82	KX920063.1
HA	1,856	42.7	B/Pennsylvania/60/2016	99.73	CY215290.1
NP	1,815	42.4	B/Hawaii/39/2017	99.89	CY249111.1
NB and NA	1,528	42.0	B/swine/Hong Kong/2850/2016	99.87	MG692775.1
M1 and BM2	1,153	38.7	B/South Carolina/09/2016	99.83	KX920051.1
NEP and NS1	1,066	40.2	B/swine/Hong Kong/2850/2016	99.72	MG692778.1

**FIG 1:**
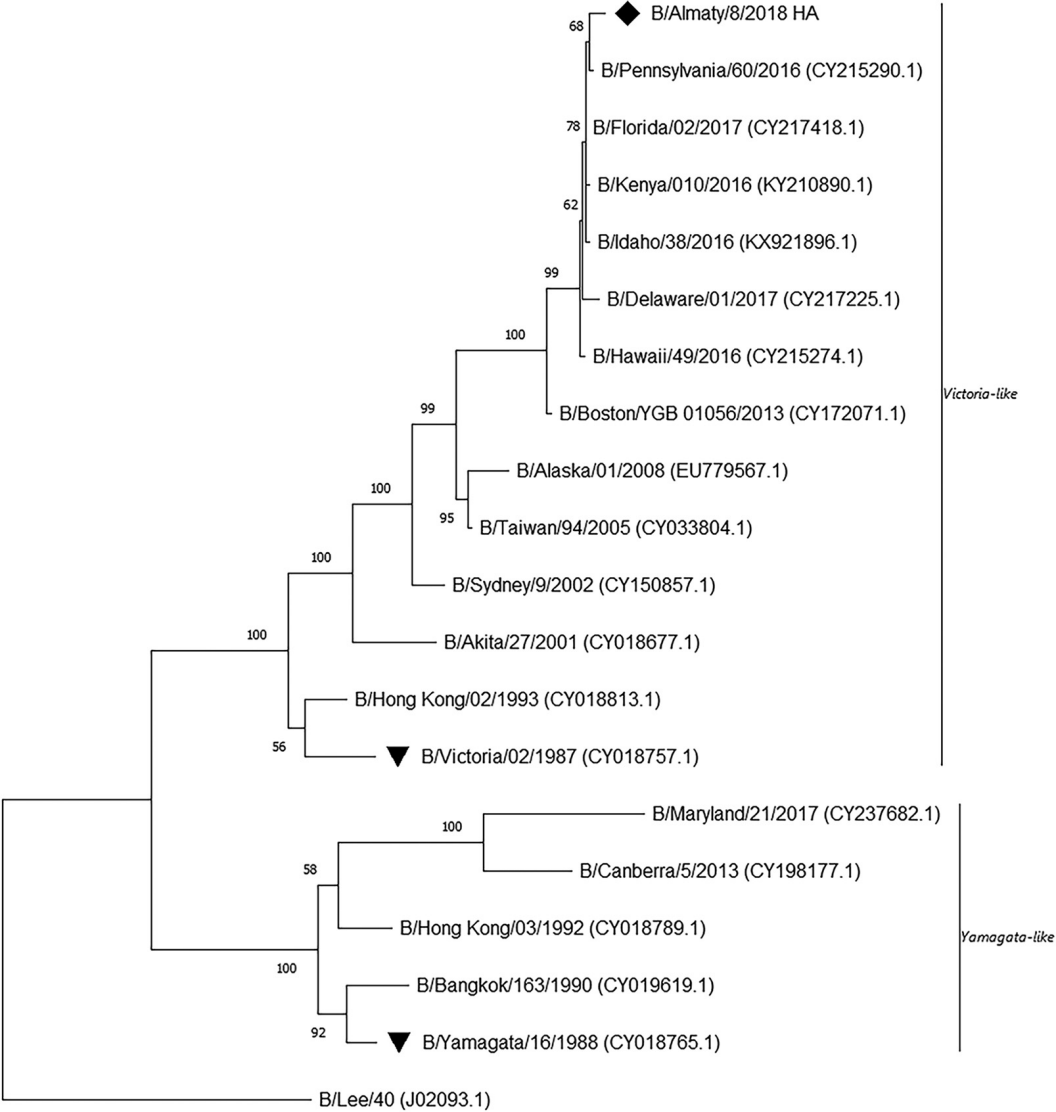
Phylogenetic tree of the hemagglutinin gene of influenza B viruses circulated around the globe. More detailed information about the phylogenetic tree is provided in the text. The Kazakhstan strain of influenza B virus is labeled with a black diamond. The reference strains of the influenza B virus evolutionary lines are labeled with black inverted triangles.

A phylogenetic tree at the nucleotide level for the hemagglutinin gene was constructed using the neighbor-joining method and the Tamura-Nei model (6) in MEGA v11.0 (7) (Fig. 1). The percentages of replicate trees in which the associated taxa clustered together in the bootstrap test (1,000 replicates) are shown next to the branches in Fig. 1 (8). The evolutionary distances were computed using the Tamura-Nei method (6) and are in the units of the number of base substitutions per site. The rate variation among sites was modelled with a gamma distribution (shape parameter, 3). This analysis involved 49 nucleotide sequences. There were a total of 1,779 positions in the final data set.

As can be seen from the phylogenetic tree, the data on the greatest similarity of the Kazakhstan isolate to similar strains isolated in the United States in 2017 were confirmed, indicating the global spread of similar strains. It was also determined that the Kazakhstan strain belongs to the B/Victoria lineage (Fig. 1).

All research components involving human subjects or other animals were conducted according to regulations under the legislation on rules for conducting biomedical experiments, preclinical (nonclinical) and clinical studies (Regulation No. 697 [12 November 2007], Republic of Kazakhstan) and were approved by the local ethics committee (approval number 2022-02-14-ICPI) of the Research and Production Center for Microbiology and Virology.

### Data availability.

The complete genome sequence of B/Almaty/8/2018 is available at GenBank under the accession numbers ON142487 to ON142494. Raw sequence reads were deposited under BioProject accession number PRJNA868912.
